# Association between composite dietary antioxidant index and handgrip strength in American adults: Data from National Health and Nutrition Examination Survey (NHANES, 2011-2014)

**DOI:** 10.3389/fnut.2023.1147869

**Published:** 2023-03-31

**Authors:** Dongzhe Wu, Hao Wang, Wendi Wang, Chang Qing, Weiqiang Zhang, Xiaolin Gao, Yongjin Shi, Yanbin Li, Zicheng Zheng

**Affiliations:** ^1^Department of Physical Education, Central South University, Changsha, China; ^2^Sports Rehabilitation Center, China Institute of Sport Science, Beijing, China; ^3^College of Physical Education and Health, East China Normal University, Shanghai, China; ^4^Department of Physical Education and Art, China Agricultural University, Beijing, China; ^5^Department of Human Health Science Research, Tokyo Metropolitan University, Tokyo, Japan; ^6^Human and Social Sciences, Chemnitz University of Technology, Chemnitz, Germany

**Keywords:** composite dietary antioxidant index (CDAI), handgrip strength, National Health and Nutrition Examination Survey, muscle strength, antioxidant

## Abstract

**Background:**

The Composite Dietary Antioxidant Index (CDAI), a composite score of multiple dietary antioxidants (including vitamin A, C, and E, selenium, zinc, and carotenoids), represents an individual’s comprehensive dietary antioxidant intake profile. CDAI was developed based on its combined effect on pro-inflammatory markers Tumor Necrosis Factor-α (TNF-α) and anti-inflammatory effects of Interleukin-1β (IL-1β), which are associated with many health outcomes, including depression, all-cause mortality, colorectal cancer, etc. Handgrip strength is used as a simple measure of muscle strength, not only is it highly correlated with overall muscle strength, but also serves as a diagnostic tool for many adverse health outcomes, including sarcopenia and frailty syndromes.

**Purpose:**

The association between CDAI and Handgrip strength (HGS) is currently unclear. This study investigated the association between CDAI (including its components) and HGS in 6,019 American adults.

**Method:**

The research data were selected from the 2011–2014 National Health and Nutrition Survey (NHANES), and a total of 6,019 American adults were screened and included. A weighted generalized linear regression model was used to evaluate CDAI (including its components) and HGS.

**Results:**

(1) CDAI was significantly positively correlated with HGS (β = 0.009, 0.005∼0.013, *P* < 0.001), and the trend test showed that compared with the lowest quartile of CDAI, the highest quartile of CDAI was positively correlated with HGS (β = 0.084, 0.042∼0.126, *P* = 0.002) and significant in trend test (*P* for trend < 0.0100). Gender subgroup analysis showed that male CDAI was significantly positively correlated with HGS (β = 0.015, 0.007∼0.023, *P* = 0.002), and the trend test showed that compared with the lowest quartile of CDAI, the highest quartile of CDAI was positively correlated with HGS (β = 0.131, 0.049∼0.213, *P* = 0.006) and the trend test was significant (*P* for trend < 0.0100). There was no correlation between female CDAI and HGS, and the trend test was not statistically significant (*P* > 0.05). (2) The intake of dietary vitamin E, Zinc and Selenium showed a significant positive correlation with HGS (β = 0.004, 0.002∼0.007, *P* = 0.006; β = 0.007, 0.004∼0.009, *P* < 0.001; β = 0.001, 0.001∼0.001, *P* < 0.001), vitamin A, vitamin C and carotenoid were significantly associated with HGS in the Crude Model, but this significant association disappeared in the complete model with the increase of control variables. Gender subgroup analysis showed that in model 3, male dietary intake levels of vitamin E, Zinc, and Selenium were significantly positively correlated with HGS (β = 0.005, 0.002∼0.009, *P* = 0.011; β = 0.007, 0.004∼0.011, *P* = 0.001; β = 0.001, 0.001∼0.001, *P* = 0.004), the rest of the indicators had no significant correlation with HGS. Among the female subjects, dietary zinc intake was significantly positively correlated with HGS (β = 0.005, 0.001∼0.008, *P* = 0.008), and there was no significant correlation between other indicators and HGS (*P* > 0.05).

**Conclusion:**

There was an association between the CDAI and HGS, but there was a gender difference, and there was an association between the CDAI and HGS in male, but the association was not significant in female. Intake of the dietary antioxidants vitamin E, selenium, and zinc was associated with HGS in male, but only zinc was associated with HGS among dietary antioxidants in female.

## 1. Introduction

The main physiological feature of sarcopenia is the progressive loss of skeletal muscle mass and strength, and the disease is considered to be a multifactorial event characterized by chronic inflammation, oxidative stress, motor neuron loss, endocrine dysfunction, nutritional imbalances, physical inactivity also exacerbates the loss of muscle strength and mass (1). It is known that oxidative stress and molecular inflammation play an important role in the decline of muscle strength and muscle mass associated with sarcopenia (2). Related studies have confirmed that the increase in chronic inflammation mediated by oxidative stress can directly induce low bone muscle strength and muscle mass (3). Many factors related to sarcopenia are not isolated, and many of their causal pathways have cross-effects or overlapping effects with oxidative stress (2).

The overproduction of reactive oxygen species (ROS) has been linked to the development of various chronic and degenerative diseases, such as cancer, respiratory, neurodegenerative, and digestive diseases. Under physiological conditions, ROS concentrations are subtly regulated by antioxidants, which can be either produced internally or supplemented externally. The combination of antioxidant deficiency and poor nutrition may make individuals more vulnerable to oxidative stress, thereby increasing the risk of adverse health reactions occurring (4). Antioxidants play a key role at the cellular molecular level, and low levels of antioxidant intake may lead to the massive production of reactive oxygen species (ROS) (5). Under normal conditions, ROS and ROS by-products are produced in the mitochondrial respiratory chain of aerobic cells, and the excessive production of ROS will generate oxidative stress (6), indirectly destroying the structure of muscle cells and inducing muscle cell disorders (7). Previous studies have confirmed that high levels of ROS can cause direct damage to macromolecules such as lipids, nucleic acids, and proteins (2). Mitochondria are the main source of ROS in skeletal muscle, and mitochondrial DNA may be particularly susceptible to oxidative DNA damage (8). The accumulation of mitochondrial and nuclear DNA damage is thought to ultimately impair muscle health, resulting in the loss of muscle cells, and decreased muscle strength (9).

Since oxidative stress is one of the important reasons for mediating the exacerbation of sarcopenia and the decline of muscle strength, it is known that dietary antioxidants can not only to reduce the level of oxidative stress in the human body and maintain skeletal muscle health, but also to improve the quality of healthy life (10–13). Relevant studies have confirmed that antioxidants such as retinol, carotenoids, and vitamin E can effectively control the level of oxidative stress to improve muscle strength (14–17). A population-based longitudinal study conducted by Sahni et al. (14) confirmed that dietary intake of carotenoids, vitamin E, and lutein+zeaxanthin, and other micronutrients were significantly and positively associated with handgrip strength. Further support for these findings was provided by Bruno et al. (18), which found a positive correlation between serum α-carotene and handgrip strength in older adults. An earlier study by Semba et al. (19) demonstrated that serum carotenoids, vitamin E, and higher handgrip strength were associated with older women. It is known that some previous studies have demonstrated the relationship between serum antioxidants and muscle strength, but most of the relevant studies have used a single class of serum antioxidants for correlation analysis. So far, few observational studies have investigated the relationship between comprehensive dietary antioxidant intake and muscle strength.

Composite Dietary Antioxidant Index (CDAI), developed by Wright et al. (20), is a composite score of multiple dietary antioxidants (including vitamin A, C, E, selenium, zinc, and carotenoids), representing an individual’s overall dietary Antioxidant intake profile. The CDAI was developed based on its combined effect on anti-inflammatory effects based on the pro-inflammatory markers Tumor Necrosis Factor-α (TNF-α) and Interleukin-1β (IL-1β), which are associated with many health outcomes, including depression, all-cause mortality, colorectal cancer, etc. (21–25).

Dietary antioxidants are known to be effective interventions for adverse health effects such as oxidative stress and chronic inflammation, but the relationship between CDAI and muscle strength is currently unclear. In this study, we investigated for the first time the independent and joint associations of CDAI (including vitamin A, vitamin C, vitamin E, carotenoids, zinc, and selenium) with handgrip strength using a cross-sectional design. Based on previous studies, we hypothesize that there may be a potential positive relationship between CDAI and HGS, and the HGS increases with CDAI.

## 2. Materials and methods

### 2.1. Design

Participants for the study were drawn primarily from the National Health and Nutrition Examination Survey (NHANES), a nationally representative population-based survey led by the Centers for Disease Control and Prevention to assess the health and nutritional status of American adults and children. The Centers for Disease Control and Prevention conducted and published data from a two-year survey using a stratified multistage probability design to obtain a representative sample of a nationally representative sample of approximately 10,000 non-institutionalized individuals across the United States. The survey protocol was approved by the Institutional Review Board of the Centers for Disease Control and Prevention (CDC) National Center for Health Statistics. Each participant agreed to sign an informed consent form.

Participants were asked questions about demographic, socioeconomic, diet, and health-related parameters and underwent medical examinations, including medical, physiological, and biochemical measurements, etc. Handgrip strength as the main variable in this study was only tested in the two cycles of NHANES 2011–2014, so it was selected as the main data source.

**FIGURE 1:**
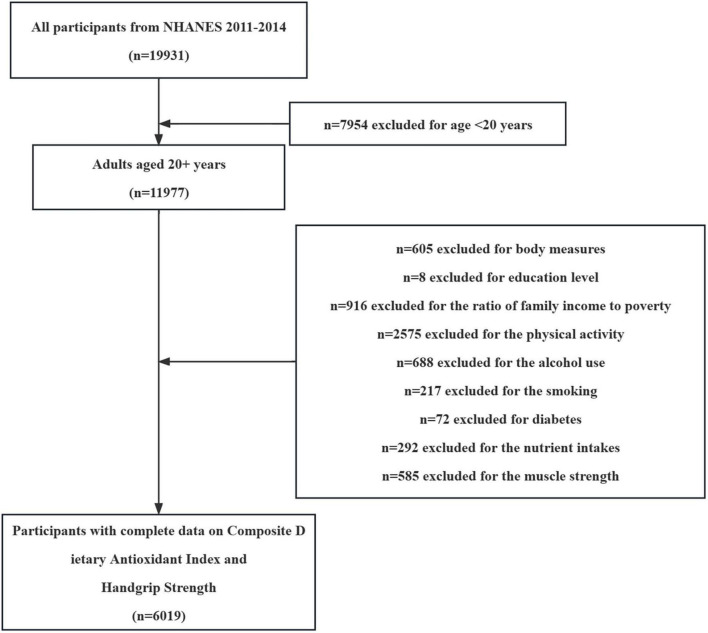
Flowchart depicting participant selection in the study.

Between 2011 and 2014, a total of 19,931 participants were sampled by the National Health and Health Administration. Our study inclusion criteria were as follows: (i) Adults over 20 years of age; (ii) Participants underwent a full handgrip strength testing process; (iii) Providing valid dietary intake data; (iv) Providing valid self-reported personal interview data (including household, sample population, and medical statistics questionnaires). After excluding those who did not meet the criteria or had missing data (13,912 people), 6,019 people were included as participants in this study (Figure 1). This study was approved by the Research Ethics Review Board of the National Center for Health Statistics.

### 2.2. Procedure

#### 2.2.1. Dietary assessment

During the NHANES investigation professional enumerators collected through two separate 24-hour dietary recall interviews and assessed as the average of the two recalls. The first recall was collected in person at a mobile inspection center, and the second interview was conducted 3–10 days later by telephone consultation, using dietary intake data for two non-consecutive days was more accurate than using single-day data (26). Participants were asked to recall details of food and drink consumed in the 24 hours preceding the interview. Six dietary antioxidant exposures of concern were studied: vitamin A, vitamin C, vitamin E, zinc, selenium, and carotenoids. Dietary antioxidant estimates do not include antioxidants obtained from dietary supplements, medications, or ordinary drinking water. To assess joint exposure from dietary antioxidant intake, we used a modified version of the Composite Dietary Antioxidant Index (CDAI) developed by Wright et al. (20, 25, 27).

Briefly, normalization was performed for each of the six dietary antioxidants by subtracting the mean from the intake of each antioxidant and dividing by the standard deviation. Next, calculate the CDAI by adding the standardized dietary antioxidant intake (21).


$$C\,D\,A\,I=\sum_{i=1}^{6}\frac{x_{i}-\mu_{i}}{s_{i}}$$

In this formula, *x*_*i*_ represents the daily intake of antioxidant; μ_*i*_ represents the mean of *x*_*i*_; over the entire cohort for antioxidant; *S*_*i*_ represents the standard deviation for μ_*i*_ (21).

#### 2.2.2. Handgrip strength testing

Handgrip strength (HGS) was measured by a Takei Dynamometer (TKK 5401; Takei Scientific Instruments, Tokyo, Japan). Participants were asked to maintain an upright posture, with their arms vertically downward, and perform handgrip strength testing with a hand-held ergometer (28). The test was repeated three times for both hands (dominant and non-dominant), with the 60 s between each measurement, taking the highest handgrip strength value for each hand. The ratio of the sum of the highest handgrip strength values of each hand to the BMI was used as the relative grip strength.

#### 2.2.3. Covariates

NHANES interviews elicit sociodemographic and behavioral characteristics as study covariates, including age, sex, race/ethnicity, education, poverty-to-income ratio, smoking, alcohol use, physical activity levels, hypertension, hyperlipidemia, and diabetes.

The rationality of covariate selection in this study was determined mainly through logical sorting and screening of previous studies. First of all, age (29), gender (30, 31), BMI (32), and education level (33) as the main demographic characteristics, have a significant correlation with muscle strength. And Hoge et al. (34) showed that higher income was associated with better physical functioning in several areas relative to PIR. Relevant human (35, 36) and animal (37, 38) studies have demonstrated significant negative effects of smoking and alcohol consumption on muscle composition and strength. Scientific physical activity has been shown to improve human health and muscle strength levels (36, 39, 40), but sedentary time has a range of adverse physiological effects on muscle strength independent of physical activity (41–43). Previous studies have confirmed that hypertension (44, 45), hyperlipidemia (46–48) and diabetes (49–51), as common chronic diseases in society, all mediate the occurrence of low muscle strength and muscle mass through different physiological pathways.

Gender (Male, Female), Education (Below high school, High school, and Above high school), Race (Black, White, Mexican, Other), BMI (< 25, 25–29.9, ≥ 30 kg/m2). Poverty-to-income ratio [PIR, *via* Calculated by dividing household (or individual) income by poverty guidelines for the survey year,< 1.3, 1.3–3.49, ≥ 3.5].

Smoking conditions are divided into three categories: never (smoked less than 100 cigarettes in life), former (smoked more than 100 cigarettes in life and smoke not at all now), now (smoked moth than 100 cigarettes in life and smoke some days or every day).

Alcohol use is divided into five categories: never (drinking < 12 times in a lifetime), former (drinking ≥ 12 times in 1 year and did not drink last year, or did not drink last year but drank ≥ 12 times in a lifetime), mild (drinking ≤ 1 times a day for female and ≤ 2 times a day for male), moderate (drinking ≤ 2 times a day for female and ≤ 3 times a day for male), and heavy (drinking ≤ 3 times a day for female and ≤ 4 times a day for male).

The NHANES survey includes the Global Physical Activity Questionnaire, which assesses time spent sitting and engaging in typical physical activity over the past week. Questions capture the amount of time spent in physical activity in all areas and intensity, including vigorous and moderate activity at work, transportation activity, and vigorous and moderate activity during leisure time. We calculated the metabolic equivalent (MET) minutes of the task using the transformation recommended by NHANES. We defined total physical activity as total MET hours per week and summed over all physical activity questions. Sedentary behavior was defined as the total amount of time spent sitting, measured in hours per day.

Diagnostic criteria for hypertension: (1) Have you been told by a doctor or health professional that you have hypertension? (2) Have you ever used antihypertensive drugs? (3) Systolic blood pressure ≥ 140 mmhg and diastolic blood pressure ≥ 90 mmhg in three blood pressure measurements are considered as hypertension patients.

Diagnostic criteria for hyperlipidemia: (1) Triglyceride (TG) ≥ 150 mg/dL. (2) Serum total cholesterol (TC) ≥ 200 mg/dL, low-density lipoprotein (LDL) ≥ 130 mg/dL, high-density lipoprotein (HDL) < 40 mg/dL (male), < 50 mg/dL (female). (3) Take lipid-lowering drugs.

Diagnostic criteria for diabetes: (1) Have you been told by a doctor or health professional that you have diabetes? (2) Glycosylated hemoglobin (HbA1c) ≥ 6.5 mmol/L. (3) Fasting blood glucose (GHLU) ≥ 7.0 mmol/L. (4) Have you ever used anti-diabetic drugs?

### 2.3. Statistical analysis

All analyzes in this study include sample weight calculation, clustering, and stratification. During the data analysis process, complex sampling weight calculation provided by the NHANES analysis guide is used, and comprehensive weight calculation and weighting processing are performed on valid sample data. The CDAI was converted into quartile groups for demographic feature description analysis, and the selected demographic feature-related indicators were expressed as the mean (standard error) of continuous variables and the percentage (%) of categorical variables. The chi-square test was used to analyze categorical variables and the One-Way ANOVA was used to analyze normal continuous variables. A weighted generalized linear regression model was used to analyze the linear relationship between CDAI (including continuous variables and quartile groups) and CDAI components (vitamin A, vitamin C, vitamin E, selenium, zinc, and carotenoids) and HGS. Model 1 is a Crude model, and model 2 adds control variables such as age, gender, BMI, education level, race, PIR, smoking, drinking, physical activity, and sedentary time on the basis of Model 1. Model 3 adds Control variables such as diabetes, hypertension, and hyperlipidemia on the basis of Model 2. Gender subgroup analyzes were further performed after controlling for covariates in weighted generalized linear models. Two-sided *P* < 0.05 was set as the threshold for statistical significance, and all analyzes were performed by R Studio version (4.2.1, USA).

## 3. Results

### 3.1. Demographics

This study included 6,109 participants (Age: 45.67 ± 0.53 years; 46.29% were female, 53.71% were male). Table 1 shows the baseline characteristics of the study population according to CDAI quartiles, gender, age, race, BMI, education level, smoking status, alcohol status, hyperlipidemia, handgrip strength, and sedentary time in different quartile groups were significant differences (*P* < 0.05).

**TABLE 1:** Demographic characteristics stratified by Quartile of CDAI (*n* = 6,019).

Characteristic	Overall	Quartile 1 (−7.585,−2.143)	Quartile 2 (−2.143,0.017)	Quartile 3 (0.017,2.712)	Quartile 4 (2.712,19.662)	*P*-value
N	6,019	1,505	1,506	1,503	1,505	
Age, years	45.67 (0.53)	45.31 (0.78)	45.85 (0.64)	47.34 (0.72)	44.10 (0.65)	**< 0.001**
Sex						**0.01**
Female	2,786 (46.29)	762 (52.03)	667 (45.21)	680 (47.49)	677 (44.59)	
Male	3,233 (53.71)	743 (47.97)	839 (54.79)	823 (52.51)	828 (55.41)	
Race						**0.003**
Mexican	637 (10.58)	139 (6.52)	166 (7.90)	175 (7.66)	157 (7.28)	
Black	1,342 (22.3)	398 (13.14)	337 (10.49)	282 (7.96)	325 (9.98)	
White	2,652 (44.06)	639 (67.85)	649 (68.66)	691 (73.02)	673 (70.75)	
Other	1,388 (23.06)	329 (12.49)	354 (12.95)	355 (11.35)	350 (11.98)	
Body mass index, kg/m^2^						**0.01**
< 25	1,931 (32.08)	461 (28.57)	456 (29.73)	489 (32.93)	525 (34.63)	
25–29.9	1,984 (32.96)	469 (32.88)	508 (34.74)	508 (34.61)	499 (35.75)	
> = 30	2,104 (34.96)	575 (38.55)	542 (35.54)	506 (32.46)	481 (29.62)	
Education level						**< 0.0001**
Above	3,742 (62.17)	813 (58.96)	897 (66.25)	1,007 (73.20)	1,025 (73.70)	
Below	1,006 (16.71)	327 (15.77)	285 (14.49)	206 (8.83)	188 (8.39)	
High school	1,271 (21.12)	365 (25.27)	324 (19.26)	290 (17.97)	292 (17.92)	
Poverty to income ratio						**< 0.0001**
< 1.3	1,874 (31.13)	567 (27.73)	471 (21.80)	394 (16.11)	442 (20.07)	
1.3–3.49	2,091 (34.74)	550 (38.14)	516 (34.52)	536 (35.32)	489 (30.58)	
> = 3.5	2,054 (34.13)	388 (34.12)	519 (43.68)	573 (48.57)	574 (49.35)	
Smoke status						**< 0.0001**
Former	1,373 (22.81)	303 (20.03)	353 (24.41)	364 (26.47)	353 (24.64)	
Never	3,426 (56.92)	807 (51.82)	831 (55.81)	883 (58.49)	905 (61.07)	
Now	1,220 (20.27)	395 (28.16)	322 (19.78)	256 (15.04)	247 (14.29)	
Alcohol status						**0.01**
Former	889 (14.77)	261 (14.86)	210 (10.65)	217 (12.32)	201 (11.32)	
Heavy	1,268 (21.07)	318 (24.58)	326 (23.22)	277 (19.34)	347 (23.20)	
Mild	2,124 (35.29)	463 (29.78)	545 (38.42)	574 (40.10)	542 (38.20)	
Moderate	1,006 (16.71)	252 (19.85)	245 (17.80)	254 (19.13)	255 (19.01)	
Never	732 (12.16)	211 (10.93)	180 (9.92)	181 (9.10)	160 (8.27)	
Diabetes						0.29
No	5,119 (85.05)	1,257 (87.44)	1,288 (89.29)	1,286 (88.65)	1,288 (90.07)	
Yes	900 (14.95)	248 (12.56)	218 (10.71)	217 (11.35)	217 (9.93)	
Hypertension						0.34
No	3,712 (61.67)	909 (63.96)	897 (63.32)	944 (65.77)	962 (66.89)	
Yes	2,307 (38.33)	596 (36.04)	609 (36.68)	559 (34.23)	543 (33.11)	
Hyperlipidemia						**0.02**
No	2,075 (34.47)	486 (30.48)	506 (33.27)	509 (32.35)	574 (37.64)	
Yes	3,944 (65.53)	1,019 (69.52)	1,000 (66.73)	994 (67.65)	931 (62.36)	
Hand grip strength, kg/BMI	2.75 (0.02)	2.62 (0.02)	2.72 (0.03)	2.75 (0.03)	2.88 (0.03)	**< 0.0001**
Composite dietary antioxidant index	0.83 (0.08)	−3.53 (0.04)	−1.07 (0.02)	1.28 (0.02)	5.91 (0.12)	**< 0.0001**
Vitamin A, μ g	644.21 (13.41)	243.38 (6.27)	448.61 (10.60)	689.36 (12.81)	1,127.64 (25.98)	**< 0.0001**
Vitamin C, mg	80.65 (2.16)	30.58 (0.83)	56.59 (1.61)	87.16 (2.44)	139.82 (5.49)	**< 0.0001**
Vitamin E, mg	9.47 (0.12)	4.29 (0.07)	7.15 (0.12)	9.72 (0.13)	15.85 (0.29)	**< 0.0001**
Zinc, mg	11.63 (0.14)	6.04 (0.09)	9.60 (0.13)	12.46 (0.13)	17.55 (0.27)	**< 0.0001**
Selenium, μ g	118.72 (1.09)	67.69 (0.96)	102.84 (1.17)	125.59 (1.45)	170.80 (3.06)	**< 0.0001**
Carotenoid, μ g	9,716.18 (230.25)	2,874.99 (90.48)	5,805.91 (217.22)	9,584.58 (199.75)	19,416.04 (535.30)	**< 0.0001**
Physical activity, MET-min/week	4,424.94 (142.66)	4,353.98 (265.66)	4,437.34 (184.09)	4,405.62 (221.61)	4,495.13 (188.44)	0.98
Sedentary time, min/day	389.90 (5.29)	373.90 (7.77)	376.16 (8.35)	398.88 (7.25)	407.34 (7.26)	**< 0.0001**

Continuous variables are expressed as mean (standard error), and categorical variables are expressed as *n* (%). Chi-square test was used for categorical variables, and analysis of One-Way ANOVA was used for continuous variables. *P* < 0.05 was set as the threshold of statistical significance and marked in bold values. CDAI quartile range: quartile 1: −7.585−2.143; quartile 2: −2.144−0.017; Quartile 3: 0.018−2.712; Quartile 4: 2.713−19.662.

In contrast, the highest CDAI quartile group tends to be younger, male, white, with a higher BMI, with higher economic level and education level. Never-smokers and mild drinkers had higher CDAI levels, and the number of people with hyperlipidemia also decreased with increasing CDAI levels.

In addition, HGS increased with the increase of CDAI level, and sedentary time also showed a trend change with the increase of CDAI level, but there was no difference in physical activity level among different CDAI quartile groups.

### 3.2. The association between composite dietary antioxidant index and handgrip strength

Table 2 shows the generalized linear regression weighted model of HGS and CDAI. In the fully adjusted model (Model 3), CDAI (continuous value) was significantly positively correlated with HGS (β = 0.009, 0.005–0.013, *P* < 0.001), and compared with the lowest quartile of CDAI, the positive correlation between the highest quartile of CDAI and HGS was more significant (β = 0.084, 0.042 ∼ 0.126, *P* = 0.002), and the trend test was also significant (*P* for trend < 0.0100).

**TABLE 2:** The association between composite dietary antioxidant index and handgrip strength.

	Model I	Model II	Model III
	β (95%CI) *P*-value	β (95%CI) *P*-value	β (95%CI) *P*-value
**Composite dietary antioxidant index**			
CDAI (Continuity Value)	0.024 (0.018, 0.030) **< 0.0001**	0.009 (0.005, 0.013) **< 0.0010**	0.009 (0.005, 0.013) **< 0.0010**
Quartile 1	Ref	Ref	Ref
Quartile 2	0.098 (0.027, 0.169) **0.0090**	−0.001 (−0.046, 0.044) 0.9620	−0.002 (−0.048, 0.045) 0.9360
Quartile 3	0.132 (0.062, 0.203) **< 0.0010**	0.051 (0.006, 0.096) **0.0300**	0.05 (0.001, 0.099) **0.0470**
Quartile 4	0.264 (0.187, 0.341) **< 0.0001**	0.083 (0.043, 0.123) **< 0.0010**	0.084 (0.042, 0.126) **0.0020**
*P* for trend	**< 0.0001**	**< 0.0010**	**< 0.0100**
**Stratified by sex**			
**Male**			
CDAI (Continuity Value)	0.024 (0.014, 0.034) **< 0.0001**	0.015 (0.007, 0.023) **0.0020**	0.015 (0.007, 0.023) **0.0020**
Quartile 1	Ref	Ref	Ref
Quartile 2	0.024 (−0.074, 0.122) 0.6170	−0.026 (−0.099, 0.047) 0.4520	−0.024 (−0.099, 0.052) 0.4900
Quartile 3	0.082 (−0.002, 0.166) 0.0570	0.058 (−0.017, 0.133) 0.1150	0.06 (−0.016, 0.137) 0.1060
Quartile 4	0.222 (0.120, 0.324) **< 0.0010**	0.13 (0.050, 0.210) **0.0040**	0.131 (0.049, 0.213) **0.0060**
*P* for trend	**< 0.0001**	**< 0.0010**	**< 0.0010**
**Female**			
CDAI (Continuity Value)	0.012 (0.006, 0.019) **< 0.0010**	0.003 (−0.001, 0.007) 0.1040	0.003 (−0.001, 0.007) 0.0890
Quartile 1	Ref	Ref	Ref
Quartile 2	0.009 (−0.073, 0.091) 0.8210	0.021 (−0.036, 0.077) 0.4390	0.017 (−0.041, 0.075) 0.5080
Quartile 3	0.075 (0.008, 0.142) **0.0300**	0.037 (−0.008, 0.081) 0.1000	0.032 (−0.014, 0.079) 0.1440
Quartile 4	0.119 (0.048, 0.190) **0.0020**	0.025 (−0.021, 0.071) 0.2550	0.026 (−0.020, 0.072) 0.2320
*P* for trend	**< 0.0010**	0.1460	0.1370

Model I (Crude): unadjusted for covariates; Model II: adjusted for gender, age, race, BMI, education level, PIR, smoking status, alcohol status, sedentary time, and physical activity level; Model III: adjusted for gender, age, race, BMI, education level, PIR, smoking status, alcohol status, sedentary time, physical activity level, diabetes, hypertension, and hyperlipidemia were adjusted. *P* < 0.05 was set as the threshold of statistical significance and marked in bold values.

Gender subgroup analysis showed that in model 3, male CDAI was significantly positively correlated with HGS (β = 0.015, 0.007–0.023, *P* = 0.002), and the trend test showed that compared with the lowest quartile of CDAI, the positive correlation between the highest quartile of CDAI and HGS was more significant (β = 0.131, 0.049∼0.213, *P* = 0.006) and the trend test was also significant (*P* for trend < 0.0100). There is no correlation between female CDAI and HGS, and the trend test has no statistical significance (*P* > 0.05).

### 3.3. The association between composite dietary antioxidant index (Components) and handgrip strength

Table 3 shows the generalized linear regression weighted model between HGS and dietary antioxidants. In model 1, all dietary antioxidants are positively correlated with HGS (*P* < 0.05), but the fully adjusted model (model 3), only showed that dietary Vitamin E, Zinc, and Selenium had a significant positive correlation with HGS (β = 0.004, 0.002∼0.007, *P* = 0.006; β = 0.007, 0.004∼0.009, *P* < 0.001; β = 0.001, 0.001∼0.001, *P* < 0.001), Vitamin A, Vitamin C, and Carotenoid were significantly associated with HGS in the Crude Model, but this association disappeared in the complete model with the inclusion of control variables.

**TABLE 3:** The association between composite dietary antioxidant index (Components) and handgrip strength.

	Model I	Model II	Model III
	β (95%CI) *P*-value	β (95%CI) *P*-value	β (95%CI) *P*-value
**Composite Dietary Antioxidant Index (Components)**			
Vitamin A	0.001 (0.001,0.001) < **0.001**	0.001 (0.001, 0.001) 0.435	0.001 (0.001, 0.004) 0.426
Vitamin C	0.001 (0.001,0.001) **0.002**	0.001 (0.001, 0.001) 0.126	0.001 (0.001, 0.001) 0.142
Vitamin E	0.021 (0.017,0.026) < **0.0001**	0.004 (0.002, 0.007) **0.006**	0.004 (0.002, 0.007) **0.006**
Zinc	0.036 (0.032,0.040) < **0.0001**	0.007 (0.004, 0.009) < **0.0001**	0.007 (0.004, 0.009) < **0.001**
Selenium	0.004 (0.003,0.004) < **0.0001**	0.001 (0.000, 0.001) < **0.001**	0.001 (0.001, 0.001) **< 0.001**
Carotenoid	0.001 (0.001,0.001) **< 0.001**	0.001 (0.001, 0.001) 0.761	0.001 (0.001, 0.001) 0.741
**Stratified by Sex**			
**Male**			
Vitamin A	0.001 (0.001,0.001) 0.108	0.001 (0.001, 0.001) 0.337	0.001 (0.001, 0.001) 0.343
Vitamin C	0.001 (0.001,0.001) 0.104	0.001 (0.001, 0.001) 0.053	0.001 (0.001, 0.001) 0.060
Vitamin E	0.01 (0.005,0.015) **< 0.001**	0.005 (0.002, 0.009) **0.009**	0.005 (0.002, 0.009) **0.011**
Zinc	0.011 (0.006,0.016) **< 0.0001**	0.007 (0.004, 0.011) **< 0.001**	0.007 (0.004, 0.011) **0.001**
Selenium	0.001 (0.001,0.002) **< 0.0001**	0.001 (0.000, 0.001) **0.002**	0.001 (0.001, 0.001) **0.004**
Carotenoid	0.001 (0.001,0.001) 0.389	0.001 (0.001, 0.001) 0.688	0.001 (0.001, 0.001) 0.658
**Female**			
Vitamin A	0.001 (0.001,0.001) 0.181	0.001 (0.001, 0.001) 0.722	0.001 (0.001, 0.001) 0.763
Vitamin C	0.001 (0.001,0.001) **0.016**	0.001 (0.001,0.001) 0.864	0.001 (0.001,0.001) 0.828
Vitamin E	0.007 (0.001,0.013) **0.022**	0.002 (−0.001, 0.005) 0.155	0.002 (−0.001, 0.005) 0.202
Zinc	0.008 (0.003,0.014) **0.004**	0.004 (0.001, 0.008) **0.008**	0.005 (0.001, 0.008) **0.008**
Selenium	0.001 (0.001,0.001) **0.017**	0.001 (0.001,0.001) 0.067	0.001 (0.001,0.001) 0.062
Carotenoid	0.001 (0.001,0.001) 0.002	0.001 (0.001,0.001) 0.836	0.001 (0.001,0.001) 0.816

Model I (Crude): unadjusted for covariates; Model II: adjusted for gender, age, race, BMI, education level, PIR, smoking status, alcohol status, sedentary time and physical activity level; Model III: adjusted for gender, age, race, BMI, education level, PIR, smoking status, alcohol status, sedentary time, physical activity level, diabetes, hypertension, and hyperlipidemia were adjusted. *P* < 0.05 was set as the threshold of statistical significance and marked in bold values.

Gender subgroup analysis showed that in model 3, male dietary intake levels of Vitamin E, Zinc, and Selenium were significantly positively correlated with HGS (β = 0.005, 0.002∼0.009, *P* = 0.011; β = 0.007,0.004∼0.011, *P* = 0.001; β = 0.001, 0.001∼0.001, *P* = 0.004), the other indicators had no significant correlation with HGS. Among the female participants, dietary intake of Zinc was significantly positively correlated with HGS (β = 0.005,0.001∼0.008, *P* = 0.008), while other indicators had no significant correlation with HGS (*P* > 0.05).

## 4. Discussion

In this nationwide cross-sectional survey based on NHANES, we found a positive correlation between CDAI and HGS, and gender subgroup analysis showed that this phenomenon was more significant in the male population. In addition, the study confirmed that there was a positive correlation between the dietary antioxidants vitamin E, zinc, selenium, and HGS, and gender subgroup analysis showed that there was a positive correlation between dietary antioxidants vitamin E, zinc, selenium, and HGS in male, but only a significant positive correlation was found between zinc and HGS in female.

So far, few studies have reported the association between CDAI and HGS. This study is the first cross-sectional survey using a composite dietary antioxidant index to explain changes in the level of HGS. In addition, our study also conducted an association analysis between the representative components of CDAI and HGS. This study confirmed that dietary antioxidants vitamin E, zinc, and selenium all have a positive contribution to the improvement of HGS. The following related studies provide further support for the findings of this study.

A cross-sectional survey from Italy confirmed that low plasma selenium concentrations were associated with poor skeletal muscle strength in community-dwelling older adults, but whether dietary selenium supplementation can attenuate age-related declines in muscle strength remains inconclusive (52). Consistent with the study by Perri et al. (53), which demonstrated that lower selenium intake is common in older populations and is associated with poor musculoskeletal function, this study did not find an association between selenium intake and HGS in female (similar results were also found in our study). A systematic review by van Dronkelaar et al. (54) confirmed that an important potential antioxidant for the adverse health physiological effects associated with sarcopenia is “selenium,” which showed a positive relationship between selenium and muscle strength in four representative studies included in the article (54–59). The above studies are consistent with the results of this study. Related research theory believes that lower serum selenium levels will limit the synthesis of selenoproteins in skeletal muscle, and lower selenoproteins in skeletal muscles will increase oxidative stress and oxidative damage in muscle tissue, decreased muscle strength through the upregulation of inflammatory cytokines, whereas lower concentrations of selenium in serum directly induced higher concentrations of biomarkers of oxidative stress (F2-isoprostane) (60–64).

Yongjae et al. (65) found that lower levels of serum vitamin E were associated with lower HGS through a cross-sectional survey. It was recommended that people with lower HGS levels be supplemented with vitamin E to prevent the risk of sarcopenia, but gender subgroup analysis showed no significant association between vitamin E intake and HGS in women. Richard et al. (19) confirmed in a survey of sarcopenia among community women that vitamin E was independently associated with HGS and knee muscle strength, confirming the hypothesis that oxidative stress is associated with sarcopenia in the elderly. Oxidative stress is believed to play an important role in the aging rate and is an important component in the aging process and pathological pathway. Vitamin E can effectively be an antioxidant and regulate the REDOX balance in the body (66, 67). A cross-sectional survey by Welch et al. (68) found a positive association between dietary vitamin E and lean body mass index, further supporting the association between vitamin E and skeletal muscle health, but similar to our findings in this study, the study concluded that vitamin E intake is not related to female HGS. An earlier study by Benedetta et al. (69) provided valuable evidence that low vitamin E concentrations were associated with the subsequent decline in physical function in a population-based sample of community-based older adults. Despite the controversial views (55, 70), we can still explore the potential contribution of vitamin E to the improvement of muscle strength through the existing relevant theory (71). Vitamin E is a fat-soluble vitamin that exerts antioxidant properties by reducing oxidative damage by scavenging reactive oxygen species (ROS) and enhancing cellular antioxidant capacity. Vitamin E, a tocotrienol-rich fraction (TRF), was found to reverse myoblast senescence in a stress model, suggesting a potential therapeutic effect of vitamin E on muscle cells (72, 73). Relevant animal experiments have demonstrated that ROS and inflammation can directly aggravate the deterioration of muscle atrophy, and vitamin E can reduce the pro-inflammatory cytokine response, regulate the inflammatory response, and improve muscle mass and strength by inhibiting the increase of NF-κB (inflammatory transcription factor) and its chemokines (72–81). In addition, studies have found that mitochondrial dysfunction and apoptosis are closely related to ROS and muscle atrophy. Vitamin E alleviates the effects of hypoxia on mitochondrial function and apoptosis signaling pathway, further reducing apoptosis under stress conditions and thus preventing muscle atrophy and muscle strength decline (82–85). It can be known that vitamin E can not only play a role in the plasma membrane disorder caused by oxidative stress, and reduce muscle oxidative damage, but also play an important role in preventing chronic inflammation and non-oxidative apoptosis.

Research on the relationship between dietary zinc and muscle strength is still relatively scarce. In a recent animal experiment, it was found that reducing the dietary intake of antioxidants such as vitamin A, E, B6, and zinc in aged mice led to a decrease in oxidative capacity, and had a major impact on muscle health by reducing muscle function and physical activity in mice (86). A cohort survey by Nishikawa et al. (87) found that serum zinc concentration was positively correlated with HGS in patients with chronic kidney disease, and zinc deficiency patients with chronic kidney disease had a higher susceptibility to sarcopenia. The study by Xu et al. (88) found that the serum zinc content of people without sarcopenia was much higher than that of people with sarcopenia and was positively correlated with relative skeletal muscle mass index (RSMI), indicating that zinc and iron may play an important role in the development and progression of sarcopenia. As we all know, zinc, as an essential trace element, is crucial to human growth and development, immunity, metabolism, and other physiological functions. Zinc deficiency not only leads to immune function suppression but also regulates the release of inflammatory cytokines (89–97). Although there is currently a lack of clear physiological mechanism research to provide a sufficient explanation for the findings of this study, the impact of zinc on skeletal muscle health may be supplemented by anti-inflammatory effects. The concentration of zinc in monocytes can activate or inhibit the release of pro-inflammatory cytokines, and lower serum zinc concentrations will lead to immune function suppression, which in turn stimulates the release of pro-inflammatory factors such as TNF-α and IL-6, while a large number of studies have shown that chronic inflammation mediated by inflammatory factors or stress directly leads to lower muscle strength and muscle mass, leading to sarcopenia over time (54, 87, 89, 91, 98).

In this study, vitamin A, C, and carotenoids were not significantly associated with HGS. Bruno et al. (18) confirmed that serum α-carotene was positively correlated with muscle strength in the elderly, but did not find an association between carotenoids and the HGS of the elderly. Recently, a prospective cohort study found that vitamin B12 intake improved muscle strength loss in elderly people with type 2 diabetes, but vitamin A intake was not associated with muscle strength (99). The findings of Li et al. (48) are consistent with the above studies, which found that vitamin A was associated with adult quality of life, but it was not associated with muscle strength in different age groups (100). A cohort study by Sahni et al. (14) found that dietary vitamin C intake was not associated with HGS and gait speed in adults, and the study argues that previous studies have focused more on baseline concentrations of serum antioxidants (carotenoids, vitamin C, E, etc.), but the concentration of serum antioxidants will fluctuate greatly over time (especially the intake of vitamin C and carotenoids is prone to seasonal changes), compared with a single blood test, additional valuable information can be provided through dietary nutrition surveys (100). The above studies provide support for the results of this study. Controversial research views can be explained by the differences in the relevant research design, participants, and the inclusion of control variables (14). However, this study is consistent with previous research suggesting that dietary antioxidant intake is helpful in improving muscle strength.

This study is the first to explore its potential association with muscle strength through a new composite dietary antioxidant index. Based on previous studies, we added control variables such as physical activity level and sedentary time that may affect the research results. In addition, NHANES has further improved the objectivity of the research results based on its survey methods, personnel professionalism, experimental control, and scientific data collection and other factors. The index for evaluating muscle strength in this study did not use the commonly used absolute value of HGS, but a more accurate relative value of HGS for correlation analysis (28).

The NHANES database is limited in its investigation of nutrients in dietary recalls. Dietary antioxidant intake has traditionally been assessed using the Dietary Antioxidant Quality Score (DAQS) (6). DAQS shows the combined antioxidant capacity of vitamins A, C, E, Se, Mn, and Zn in six grades ranging from very poor (0) to high antioxidant quality (6). The score relates to the ratio of the daily intake of these nutrients to the corresponding recommended intake. However, this section ignores secondary plant compounds (101). In addition, authors studying the relationship between DAQS and major OS biomarkers did not report any significant relationship, namely with urinary F2-is prostaglandin, PGE-2, or other OS or inflammatory markers (101). The CDAI contains similar nutrients to DAQS but uses a different calculation method derived from the Dietary Antioxidant Index originally developed by Wright et al. (20). The latter index combines the intake of vitamins A, C, E, carotenoids, zinc, and selenium. Scores are based on principal component analysis and summary construction of retained principal component scores. Carotenoids include α-β-and γ-carotene, as well as β- cryptoxanthin, lutein, zeaxanthin, and lycopene. Vitamin E consists of α- and β-tocopherol and α- and γ-tocopherol. Moreover, studies have confirmed that CDAI is significantly associated with many adverse health effects (13, 22–24, 27, 102). Therefore, compared with traditional dietary antioxidant indexes, CDAI has proved its application advantages and effectiveness in epidemiological studies. According to the main purpose of this study and the structure of this paper, no other dietary antioxidant indexes were included for horizontal comparison.

However, there are still some limitations in this study. (1) The evaluation indicators used in this study are more obtained through subjective questionnaires rather than objective measurement indicators of blood biochemistry; (2) Although individual dietary recall in NHANES is conducted using two separate 24-hour dietary recall interviews, measurement error cannot be avoided; (3) This study tried to control social confounding factors that may affect CDAI and muscle strength, but based on source data and existing theoretical limitations, it is not possible to include all control variables that might have influenced the results of the study. (4) The gender difference between dietary antioxidants and HGS in this study is supported by relevant studies, but no effective basic theoretical support has been found for the reasons and mechanisms of this result, it is necessary to carry out further research on the relevant physiological mechanisms in the future.

## 5. Conclusion

In conclusion, the results of this study support that there is an association between the CDAI and HGS, but there is a gender difference. There is an association between the CDAI and HGS in male, but this association is not significant in female. Intake of the dietary antioxidants vitamin E, selenium, and zinc was associated with HGS in male, but only zinc was associated with HGS among dietary antioxidants in female. Therefore, research encourages attempts to consume more dietary antioxidants to improve muscle strength.

Most of the previous studies were cross-sectional or cohort investigations, and more randomized controlled trials should be carried out in the future to further explain the physiological mechanism association between dietary antioxidants and muscle strength.

## Data availability statement

Publicly available datasets were analyzed in this study. This data can be found here: https://wwwn.cdc.gov/nchs/nhanes/.

## Ethics statement

The studies involving human participants were reviewed and approved by National Health and Nutrition Examination Survey, NCHS IRB/ERB Protocol Number: NHANES 2011–2012 (Protocol #2011-17); NHANES 2013–2014 (Continuation of Protocol #2011-17). The patients/participants provided their written informed consent to participate in this study.

## Author contributions

DW, WZ, and XG: conceptualization. DW: methodology, software, investigation, and write—original draft preparation. HW, WW, CQ, and YS: validation. DW, YL, and ZZ: data curation. WZ: writing review and editing. All authors have read and agreed to the published version of the manuscript.
